# Escalation, maintenance and abstention in oncology a new study design to identify how individual values of patients impact on the assessment of risks and benefits of novel therapeutic concepts in cases of gynaecological tumors and colorectal cancer: a study protocol

**DOI:** 10.3389/fonc.2025.1588721

**Published:** 2025-07-08

**Authors:** Susanne Theis, Jülide Senyigit, Britta Büchler, Gesa Kolck, Markus Moehler, Annette Hasenburg, Norbert Paul

**Affiliations:** ^1^ Department of Obstetrics and Gynegology, University Medical Center of Johannes Gutenberg University Mainz, Mainz, Germany; ^2^ Institute of History, Philosophy, and Ethics of Medicine, Johannes Gutenberg University Medical Center Mainz, Mainz, Germany; ^3^ Insitute of Medical Biostatistical, Epidemiology and Informatics (IMBEI), Johannes Gutenberg University Medical Center Mainz, Mainz, Germany; ^4^ Department of Internal Medicine I, University Medical Center of Johannes Gutenberg University Mainz, Mainz, Germany

**Keywords:** ethical concepts, values, participation, individual goals, maintenance, abstinence

## Abstract

Being diagnosed with cancer is a life-altering event that profoundly reshapes an indivdual’s biography, especially in terms of temporality and physicality. Modern treatment approaches, such as maintenance therapy or therapeutic abstinence, often conflict with patients’ desire for a radical fight against the disease. Decision-making about therapeutic goals is shaped by the tension between medical-scientific expertise and personal values and experiences, making it particularly challenging to align diverse preferences and norms. This study aims to analyse values in the context of the challenging human experience of cancer. We hypothesise that conventional quantitative measures, including patient-reported outcome measures (PROMs) may be insufficient to fully capture patients’ perspective on therapeutic success. To address this, we will conduct semi-structured interviews with individuals diagnosed with gynecological or colorectal cancer. Using a mixed methods approach, we aim to identify patients’ values, treatment goals, and expectations. The study’s key outcomes include: 1) an ethical analysis of values in the context of cancer experiences 2) a reconstruction of values that shape individual treatment goals and expectations and 3) a comparison between ethical concepts of successful life, patients’ situational values, and evidence-based medical preferences. Building on these insights, the study pursues three secondary objectives: 1) developing a strategy for patient-centred adaptation of clinical evaluations of treatment approaches, including escalation, maintenance and abstinence, 2) designing a well-defined clinical framework for therapeutic goal setting, and 3) integrating this framework into medical education, addressing clinical, oncological, ethical and communication competencies.

## 1 Introduction

In tumor medicine, therapeutic goals are generally set and evaluated through well-established interdisciplinary procedures, such as tumor boards, which integrate evidence-based and guideline-oriented therapeutic options.

The standard of good clinical practice in tumor medicine provides for patient-centred counselling, ensuring that patients’ hopes, fears and preferences are considered - especially in the light of legal advancements in patients’ rights and ethical principles (1–4). Advances in interdisciplinary treatment approaches, such as curative colorectal metastasis surgery (5), intensive chemotherapy for decreasing tumor burden (6), and immunotherapy for long-term maintenance in microsatellite-unstable patients, have made therapeutic decision-making increasingly complex. Physicians and patients must now navigate between escalation, maintenance or abstention as viable treatment strategies.

In addition to acute interventions in the cancer dynamics, maintenance therapies have recently gained prominence in the long-term control of cancer (7, 8). While perpetuation strategies can ensure treatment success and prevent recurrences through long-term, potentially lifelong drug administration, they also bring attention to the persistent risk posed by “dormant” tumor cells or stem cells, which may inherently threaten the durability of therapeutic outcomes (9–12). The emergence of new molecular subsets, such as MSI or B-RAF mutations (13–15), has made it possible to rapidly redirect therapy, often leading to sudden and drastic shifts in treatment plans. These rapid changes can throw patients into a rollercoaster of emotions (16, 17), as they must repeatedly adapt to new possibilities, uncertainties and evolving prognoses. Furthermore, many of these innovative options – some of which have not yet been approved - require a great deal of commitment from both physicians and patients. Physicians must be willing to continuously reassess, explain, and clarify the available choices, while patients must repeatedly engage in complex decision-making. This dynamic process underscores the need for a structured approach to redefining individual therapeutic goals in an ever - evolving oncological landscape.

In gynecological oncology (which in Germany also includes breast oncology) there are various approaches to therapy and maintenance strategies. Examples include: 1. Chemotherapy, frequently used as a combination therapy in advanced stages, which can also include maintenance therapy following first-line treatment. 2. Hormone therapy for dependant tumors, such as certain types of breast cancer or endometrial cancer, which inhibits tumor growth and is often continued as maintenance therapy after chemotherapy. 3. Immunotherapy, including checkpoint inhibitors such as Pembrolizumab and Nivolumab, which are used either in combination therapies or as maintenance treatments. 4. Targeted therapies, which focus on certain molecular properties of tumors such as PARP-inhibitors (e.g. Olaparib, Niraparib) for BRCA-mutated cancers. These targeted combination therapies can be incorporated into escalation therapies, and in the same cases, chemotherapy can be intensified. Many patients are often encouraged or given the opportunity to take part in clinical trials to test the efficacy of other new therapies or combination therapies beyond current standard treatments.

Treatment of colorectal cancer (CRC) also includes various approaches to therapy and maintenance strategies. Like in gynecological and breast oncology they are tailored to tumor stage, molecular characteristics and individual patient factors. Examples for CRC include: 1) Chemotherapy as a fundamental component, particularly in advanced or metastatic stages which can also include maintenance therapy to prolong disease control and delay progression. 2) Immunotherapy, including checkpoint inhibitors such as Pembrolizumab particularly in later therapy lines for patients who have already been treated. 3) Targeted therapies like EGFR inhibitors such as Cetuximab oder Panitumumab depending on molecular subtype (presence or absence of RAS, BRAF, or HER2 mutations, as well as microsatellite instability (MSI) status). 4) Locoregional treatments like radiofrequency ablation (RFA) or selective internal radiation therapy (SIRT) in selected patients, for instance limited liver or lung metastases. Of course, patients are also motivated to participate in studies in the field of CRC oncology to provide access to innovative therapies or novel combinations that go beyond the current standard of care.

A defining aspect of cancer is that it fundamentally changes one’s biography in terms of temporality, corporeality and capabilities - also present in healthy individuals (18). Frequently, this unexpected and often radical challenge to one’s former life seems to require equally radical counter-measures in the form of aggressive surgery, radiation and chemotherapy. Dealing with cancer is often seen as a battle to be bravely fought to the end or successfully endured as a survivor. In the fight against the “hostile” cells, this widespread socio-cultural perception assumes (implicitly or explicitly) a maximum escalation of therapy. However, this assumption runs counter to modern concepts of maintenance therapy or therapeutic abstinence, not only in cases of a poor prognosis, but also when the prognosis is favorable. Little is known about how patients’ self-concepts, values - both positive and negative - evolve during the transition from usually initially escalated tumor therapy to maintenance therapy or purely aftercare. This knowledge gap is partly due to the unstructured nature of discussions about personal values and goals in the clinical context. Consequently, an important distinction is often highly blurred: that between negotiable first-order desires (i.e. situational, often reactive spontaneous desires) and non – negotiable higher-order desires (i.e., desires that are themselves related to reflected value attitudes) (19). However, this distinction is clinically significant in several ways:

1. Participatory decision-making: Effectively resolving cognitive dissonance - such as the misinterpretation (false positive or false negative) of evidence - based information - requires not only good communications skills, but also a deeper understanding of mechanisms of how value-based attitudes and decisions emerge and take shape (20, 21). This is a critical consideration in the establishment of communication training programs for clinical teams.
2. Long-time treatment adaptation: in the context of maintenance therapy, patient consent should not be viewed as a one-time agreement to procedure specific treatment plan. Instead, treatment approaches should remain flexible, adapting to both the evolving course of the disease and the dynamic nature of the patients’ values and self-concepts. A successful treatment is never exclusively a purely medical success; for patients, it must also support their ability to participate in social life. To better understand the normative preconditions of superordinate and individual concepts of social participation and to make them the venture points of physician- patient relationship, is therefore essential.
3. Integrating individual and situational values is decision-making: a participatory and dynamic decision-making process must account for patients’ individual and situational values. If individual values and context-specific expectations lead to a decision against a medically preferred course of action, the ability to differentiate between first-order desires and higher- order desires is essential. By projecting the qualitative study results onto a matrix of patient- oriented outcomes (patient-oriented outcome measures (PROM)), we aim to analyze the medical preference, practical rationality and patients’ lived experiences.

Despite the existence of clinical (medical) ethics counselling in most university hospitals, a gap remains between theoretical ethical frameworks and their practical application in everyday clinical settings. Clinical ethics should be understood as a practice that is subject to the same criteria of quality as any other kind of clinical practice. Defining clinical ethics as applied ethics in a particular field requires that the application and the resulting measures be justified by normative theory. However, clinical ethics usually relies on mid-level principles that aim for broad generalizability, ensuring argumentative robustness. This approach, while theoretically sound, can sometimes be at odds with the complexities of individual clinical cases. To address this, we propose the development of clinically operationalizable, ethics-based decision-making strategies that bridge this gap between theory and practice.

The study follows an integrative particularist approach, proposing health-related social participation as a goal-directed (teleological) normative framework for reconciling particular norms (22). The development of the concept is especially relevant, as decision- making is fundamentally about weighing the benefits and risks of medical interventions in order to maximize patients’ ability to engage in social life. By reconstructing individual values and norms in the qualitative phase of the study and lining them to clinical outcomes via PROMs, we aim to contextualize individual and professional values and norms underlying therapeutic decision-making.

We thus suggest giving increased attention to qualitative research and mixed-methods approaches as a theory-driven (23) tool for analyzing the value systems that shape goal-setting and treatment evaluation in physician-patients relationship, particularly in gynecological and gastroenterological oncology. In particular, recourse key focus of our approach is higher-order desires/values, i.e., to such ideas of a successful life, which in turn are based on reflected normative categories (health, social participation, realization of self-concepts, development or preservation of identity). In this context, standard PROMs do not distinguish between first-order and higher-order desire, nor do they accurately capture broader concepts of successful life beyond (semi-) quantitative surrogate markers for social participation. In this respect, the present project is expected to contribute to the refinement of decision-making strategies in tumor medicine, especially in gynecological tumors and colorectal carcinoma.

The tumor entities were deliberately chosen in order to be able to draw the broadest possible conclusions from the data. With this selection, we try to compare “younger women” [breast cancer mean age of onset 65 years but 15% of cases occur in women < age of 50 years (24), cervical carcinoma mean age of onset 53 years, carcinoma *in situ* 35 to 40 years (25), ovarian cancer mean age of onset 68 years (26)] with “older men” [mean age of onset CRC for men: 72 years (27)] in order to be able to demonstrate differences in values and the associated therapy objectives in the later planned comparison. This also explains why we explicitly include breast cancer patients, as this type of tumor can occur in relatively young patients (15% > 50 years at first diagnosis).

Current research in this area identifies the complex interplay between expert knowledge and patient interpretation, revealing that the experience of cancer is also shaped by professional biomedical (oncological) narratives to a far greater extent than previously assumed. This study seeks to deepen our understanding of this phenomenon and its implications for patient-centered care.

## 2 Materials and equipment

### 2.1 Interviews

- Semi- structured interview template.
- Tape recorder.

### 2.2 Coding/data evaluation

- MAXQDA Analytics Pro.

## 3 Methods

### 3.1 Study design and setting

This study is designed as a prospective clinical trial employing a mixed-methods approach, integrating qualitative and empirical research methodologies. It will be conducted at the Institute for History, Theory, and Ethics of Medicine at Johannes Gutenberg University Mainz, Germany, in collaboration with the Department of Gynecology and Obstetrics and the Department of Internal Medicine at the University Medical Centre of Johannes Gutenberg University Mainz.

As part of this prospective clinical trial, we will conduct semi-structured interviews with 40 patients—20 with an initial diagnosis of a gynecological tumor and 20 with colorectal carcinoma—who are either transitioning to or currently undergoing maintenance therapy. The study aims to reconstruct patients’ values, individual therapy goals, and their positive and negative expectations regarding social participation, identity, and self-concept.

The approach of the study presented refers to the concept of grounded theory, which is a qualitative research method aiming to develop theories that are grounded in systematically gathered data that are analyzed (28). What is special about this method, is, that data collection and analysis happen simultaneously (29). Early analysis guide further data collection – the theoretical sampling (28, 29). Data collection continues until no new concepts emerge (theoretical saturation) (30, 31).

Nevertheless, at the beginning of the study planning process, statistical consultations were held with the university’s statistical advisory office. In the end, a statistical calculation was deliberately omitted due to the mentioned approach of grounded theory and qualitative character of the interviews.

The typical number of interviews in studies using grounded theory ranges between 20 and 30 (30, 31). The decisive factor is not the number itself but the achievement of theoretical saturation. With the planned number of 40 interviews, we are therefore above the usual number and can expect a theoretical saturation and as well as meaningful results.

By employing semantic network analyses using MaxQDA 2024, we will analyze these relatively small yet highly data-rich samples to generate novel insights into the values, motivations, and expectations that shape physician - patient interactions from the patient’s perspective. This approach will contribute to a deeper understanding of the factors influencing patient engagement and decision-making in clinical settings.

### 3.2 Recruitment

Patients will be recruited at the onset of their (maintenance-) treatment plan, after the interdisciplinary tumor conferences at each hospital have finalized the treatment schedule. Written informed consent will be obtained from all participants prior to inclusion and the baseline visit. Patients have the right to withdraw their consent and discontinue participation in the trial at any point, without any consequences. The study will adhere to the German data protection regulations (DGSVO).

Furthermore, there is no treatment that could be prescribed randomly. The study is not an interventional study. The treatment of the patients is independent of participation in the study and does not change as a result. All patients treated during the recruitment period who meet the inclusion criteria can participate in the study and thus in the same partially standardized interview. Nevertheless, care will be taken in the selection of subjects to ensure that approximately 10 patients per tumor entity are in primary therapy and 10 in maintenance therapy or at the transition to a decision.

### 3.3 Eligibility criteria

Adult female patients (> 18 years) with initial histologically confirmed gynecological cancers (ovarian, breast, cervical or uterine) as well as adult male and female patients (>18 years) with histologically confirmed colorectal cancer are eligible to participate in this study. Eligible patients must be capable of giving consent and not be undergoing treatment for a psychiatric diagnosis. Patients of advanced age, who are treated based on gerontological oncology assessments and age-appropriate treatment goals, will be excluded from the study. Potential study participants will be approached by the attending physicians, who will provide them with detailed information about the study if they express interest in participating (see **Table 1**).

**Table 1:** Inclusion- and exclusion criteria.

Gynaecological tumors		Colorectal tumors	
Inclusion criteria	Exclusion criteria	Inclusion criteria	Exclusion criteria
Female	Treatment for psychiatric diagnosis	Male and female	Treatment for psychiatric diagnosis
Age > 18	advanced age who are treated on the basis of gerontological oncology assessments and age-appropriate treatment goals	Age > 18	advanced age who are treated on the basis of gerontological oncology assessments and age-appropriate treatment goals
Histologically confirmed genycological cancer (ovarian, breast, cervical, uterine)		Histologically confirmed colorectal cancer	
Capable to give consent		Capable to give consent	

### 3.4 Baseline characteristics

For eligibility screening and baseline assessment, data of potential trial participants will be recorded, including full oncologic history, full medical history, concomitant therapies as well as inclusion and exclusion criteria. Once recruited for the trial, all participants will be asked to complete a baseline questionnaire concerning social setting and education.

### 3.5 Study intervention

All participants will take part in a semi-structured interview at the beginning of their (maintenance-) therapy. This is performed by one of the trained study physicians. The transcribed interviews are processed pseudonymously.

### 3.6 Adherence

To increase compliance, all participants are offered a personal study booklet summing up information about background and aims of the project as well as contact data of the study physicians and the director of the study. Individual study-specific questions can be discussed with a medical doctor at any time during the study in person or via electronic communication.

### 3.7 Methods against bias

#### 3.7.1 Selection bias

Potential trial eligibility will be assessed by study physicians, and all patients will be approached consecutively for trial participation. Final eligibility will be determined during an initial consultation for obtaining informed consent. To mitigate selection bias, tumor types have been carefully chosen to ensure a representative sample.

#### 3.7.2 Detection bias

All outcomes will be predefined, and the majority of outcome parameters are assessed by clear definitions that minimize ambiguity. Subjective outcomes will be documented directly by the participants themselves, ensuring they remain independent of study staff influence. To enhance reliability, interpretative tools and semantic networks analysis will be validated in regular meetings involving contributing researchers and physicians.

### 3.8 Advice on qualitative methods

Good clinical practice, especially within a mixed-methods framework and following a “grounded theory” approach, will be ensured through collaboration with the Institute for Medical Biostatistic, Epidemiology and Informatics (IMBEI) at the University of Mainz. A preliminary consultation was conducted before data collection, and additional meetings will follow throughout the study to support data analysis and interim evaluations.

### 3.9 Data monitoring, interim analysis and trial termination

Data will be collected using a personalized case report form at the time of inclusion, and entered into a secure, protected database. Only authorized members of the study team will be allowed to enter, store and access participant data. Paper-based records will be stored at the department of obstetrics and women’s health at the University Medical Center Mainz as part of the clinical patient documentation. Digital data will be encrypted and stored on the approved state-based SEAFILE-Server. The study is considered to have a very high safety profile, as the intervention consists solely of a semi-structured interview. Any psychological distress that may arise from dealing with personal diagnosis can be discussed with a study doctor at any time. Participants can also be promptly referred to the psycho-oncology department at the University of Mainz if needed.

Participants may withdraw from the trial at any time without facing any negative consequences by revoking their consent. Additionally, the investigator may decide to discontinue a participant’s involvement for medical reasons at any point during the study.

## 4 Anticipated results

### 4.1 Project objectives and design

The presented prospective study, using a mixed-method approach aims to achieve three key objectives: 1) the establishment of communication training programs for clinical teams, 2) a deeper understanding of the normative preconditions of both overarching and individual concepts of social participation, integrating them as a core aspect of the of physician-patient relationship, 3) an analysis of PROMs, medical preferences, and practical rationality in their real-world, lived experience context.

### 4.2 Outcome measures

The primary outcome of the study is to understand the key factors that enable participatory ethical goal setting in the treatment of gynaecological and colorectal tumors. This includes an ethical analysis of values in the context of challenging and/or traumatising human experiences of cancer. In the context of translational clinical ethics, a reconstruction of values, individual treatment goals and realistic or misrepresented expectations- both overly optimistic and pessimistic- will facilitate a more individualised definition of therapeutic goals. This process will naturally result in particular, context-dependent norms and values that must be weighed against broader, more universal ethical principles. Well- founded ethical concepts of successful life will be used as a foundation for a more general justification of personal values, individual treatment goals, and expectations in relation to evidence-based (medical-scientific) expert preferences.

There is a paucity of data on participatory goal setting in gynaecological and colorectal cancers. Therefore, a wide range of secondary endpoints will be evaluated in future confirmatory trials. Particularly in the light of increasingly individualised oncological treatments, the establishment of a strategy for patient-oriented adaptation of the clinical evaluation of therapeutic concepts such as escalation, maintenance or abstinence is increasingly in need. Another secondary outcome measure is the modelling of a well-structured clinically applicable strategy for therapeutic goal setting. Finally, translational ethics is characterised by the research-based generation of knowledge, its application and its dissemination. Therefore, an essential secondary outcome of this study is the integration of its findings into medical education - both at undergraduate and postgraduate levels - as well as continuing medical education (CME), encompassing oncological, ethical, and communication-based competencies.

## 5 Discussion

### 5.1 Strengths

To our knowledge, this is the first prospective clinical trial using a mixed-methods approach, integrating qualitative and empirical research to investigate participatory goal setting in patients with gynecologic and colorectal cancers. With a planned sample size of 40 patients, the study is comparable to other clinical trials with similar qualitative designs. The broad eligibility criteria allow for the inclusion of patients with diverse oncological histories, encompassing various gynecological and colorectal cancers. This inclusivity enhances the generalizability of findings to routine clinical practice. Additionally, the study design - requiring only a single interview and aligning recruitment with patients’ scheduled clinical visits - facilitates high compliance rates.

### 5.2 Limitations

The inclusion of different tumor types and corresponding therapeutic strategies enhances the generalizability of the study. However, this approach also introduces greater heterogeneity in trial results. Despite this variability, these findings are important for generating new hypotheses, detecting meaningful endpoints and refining sample size estimations for future trials. Nevertheless, the data might be of limited applicability for some cancer types or treatment regimes due to the distribution of included patients.

## 6 Summary

Decisions about therapeutic goals are caught between medical-scientific expertise on the one hand and life-world judgements and values on the other. This makes it particularly challenging to reconcile different preferences and norms. To address this, we propose a mixed methods approach to identify participants’ values, individual treatment goals, and expectations concerning individualized gynecologic and colorectal cancer therapy from an ethical point of view.

In the long term, our objectives are:

1. Establishing a strategy for patient-centred adaptation of clinical evaluation of therapeutic concepts of escalation, maintenance or abstinence.
2. Developing of a well-operationalised clinical strategy for therapeutic goal setting.
3. Integrating this strategy into medical education by incorporating its oncological, ethical, and communicative aspects into undergraduate, postgraduate, and continuing medical training.

We are convinced that this approach is a valuable addition to the already highly specialized tumor therapy for patients as well as for their physicians in everyday clinical practice.

## Data Availability

The original contributions presented in the study are included in the article/**Supplementary Material**. Further inquiries can be directed to the corresponding author.
