# Supplementary material for: Escalation, maintenance and abstention in oncology a new study design to identify how individual values of patients impact on the assessment of risks and benefits of novel therapeutic concepts in cases of gynaecological tumors and colorectal cancer: a study protocol
Source: Front Oncol. 2025 Jul 8;15:1588721. doi: 10.3389/fonc.2025.1588721 (PMC12287738; doi:10.3389/fonc.2025.1588721)
Supplement: Supplementary file 1 [file DataSheet1.pdf]

Interview guide for participants

Guiding research questions:

1. How do patients with oncological diseases experience their illness, as well as the consequences of therapy and the physical and psychosocial burden of therapy?
2. Which individual therapy goals are important to patients?
3. What positive or negative expectations do patients have in terms of social participation, identity and self-concept?

Preliminary remarks:

- The guideline was developed and adapted on the basis of the guideline and initial empirical experiences from the „Illness experience of children, adolescents and young adults in the FORTEe project “(Institute for History, Theory and Ethics of Medicine University Medical Center Mainz).
- The exact wording of the questions should be geared to the age, stage of development and state of health of the participants.
- These are semi-structured guided interviews and are merely an overview of possible questions. The exact procedure and the „completeness “of the questions to be asked depend on the interview partners and their priorities.
- Square brackets indicate terms that need to be adapted to the interviewees and their individual situation.

Information phase

Groups	Topics and interview questions	Comment/ Explanation
All	Information about:<br>· Topic, aim, procedure of the interview<br>· Explanation of the interviewer's behaviour (taking notes, any necessary follow-up-questions)<br>· Voluntary nature and option to pause or terminate at any time<br>· Questions possible at any time<br>· Unanswered questions?<br>· Ask for consent to audio recording (can be recorded on this)	Creating a pleasant, informed dialogue situation.<br><br>The information phase does not have to be repeated in its entirety as long as it is clear that the participant is well acquainted with the procedure.

Entry phase

Groups	Topics and interview questions	Comment/ Explanation
All	· How are you doing today?<br>· Can you tell me what you have experienced/what has happened since we last saw each other?	Introductory question that is as open as possible, encourages people to tell their story and enables them to start a conversation.

Main phase, topic area 1: Physical experience of illness

Groups	Topics and interview questions	Comment/ Explanation
Breast Cancer, Colorectal Cancer	· A symptom that has already been discussed, a side effect of therapy or a visible medical object can be used as a starting point for the transition, e.g: You have already told me […]. How do you feel about that?<br><br>1a) <u>Physical effects due to illness</u><br>· I would like to know more about the [symptoms] of your illness. Can you tell me what [symptoms] your illness started with?<br>o How did you feel when you had these [symptoms]?<br>o What has happened to the [symptoms] since you have been in treatment?<br>o Was there anything that helped you when you had these [symptoms]?	Existing research suggests that the topic of the physical experience of illness is difficult to deal with, so the initial question is central and should be asked as openly and narrative-stimulatingly as possible.<br><br>This question tends to require the ability to look back on one's own medical history.
Breast Cancer, Colorectal Cancer	· Can you describe how it feels physically/how you feel physically about having [cancer]?	
Breast Cancer, Colorectal Cancer	1b) <u>Side effects of therapy</u><br>· Your treatment is supposed to make you healthy again, but it also has difficult side effects.<br>o Do you find any of these side effects particularly unpleasant?<br>o Can you tell me how you feel about fatigue/how you feel about feeling tired?<br>o Is there anything that helps you to deal with these side effects?	
Breast Cancer, Colorectal Cancer	· Can you tell me about the side effects of your treatment?<br>o How are you coping with these side effects?<br>o Is there anything that helps you to deal with these side effects?	
Breast Cancer, Colorectal Cancer	· How are you coping with the fact that you are restricted by the illness or therapy and the hospitalisation?	

Breast Cancer, Colorectal Cancer	· When you think about the longer-term consequences that your treatment may have, is there an issue that is of particular concern/importance to you?	
Breast Cancer, Colorectal Cancer	1c) <u>Medical and nursing measures</u><br>· Can you tell me what examinations/treatments the doctors and nurses have already carried out on you?<br>o How was the [examination/treatment] for you?<br>o Of all the [examinations/treatments], was there one that was particularly unpleasant for you?<br>· What is it like for you when [medical/care intervention] is carried out?<br>· Was there anything that helped you to get used to […]?	
Experts	1d) What are the reasons for escalating, maintenance or withholding treatment in this patient?<br>· Did you face any particular challenges in communicating the therapy goals? If so, which ones?<br>· Do you feel sufficiently prepared for these challenges through your training/professional experience?<br>· Which specific elements of doctor-patient-communication are particularly helpful you you?<br>· Are there any particular characteristics of your patient that you have found particularly challenging or helpful when communicating?	

Main phase, topic area 2: Individual therapy goals

Groups	Topic and interview questions	Comment/ Explanation
Breast Cancer, Colorectal Cancer	2a) <u>Learn about individual therapy goals</u><br>· You have recently started your [maintenance therapy/invasive therapy]/ your therapy has finished.<br>o What is your primary goal of the therapy?<br>o What other secondary goals do you hope to achieve?	Identifying first-order goals/desires and higher-order desires
Experts	2b) What is the primary goal of the therapy?<br>· Which findings define the therapy goal?<br>· Can you make a statement about the prognosis?	

Main phase, topic area 3: Positive and negative expectations regarding social participation, identity and self-concept

Groups	Topic and interview questions	Comment/ Explanation
Breast Cancer, Colorectal Cancer	3a) <u>Positive and negative expectations regarding social participation</u><br>· The [illness itself and the] treatment of the illness naturally also has an impact on your environment, including first and foremost your family and circle of friends, but also simple acquaintances.<br>o What has changed for you socially since you were diagnosed with cancer?<br>o What has changed for you socially since you started therapy?<br>o How do you feel when you are in company?<br>o How do people in your social environment behave towards you?<br>o Do you have the feeling that your environment is behaving differently than before?<br>o Are there any restrictions due to the cancer itself or the therapy that prevent you from participating in social life?<br>o Are you afraid of not being able to participate in social life as a result of the therapy measures/illness itself?	

Colorectal Cancer	3b) Positive and negative expectations regarding own identity and self-image<br>· Would you say that relationship with your body has changed as a result of the illness?<br>· What influence does the disease have on your body image?<br>· Has your self-image changed [before and after becoming ill]? If yes, in what way?	Other issues in older men with colorectal carcinoma.<br><br>Threat to the intact unity of the body (integrity/wholeness)
Breast Cancer	3c) Positive and negative expectations regarding own identity and self-image<br>· Would you say that your relationship with your body has changed as a result of the illness?<br>· What is/was your ideal image of a woman? Has this ideal image changed as a result of the illness?<br>· What influence does the disease have on your own sense of femininity [your female identity/attractiveness/sexuality]	Other issues in younger women with breast cancer.<br><br>Role of the female breast/ female reproductive organs.
Experts	3d) Assessment of the patient<br>· How do you perceive your patient?<br>· How would you rate your patient’s satisfaction?<br>· How would you rate the patient’s co-operation in the therapy?<br>· How do you assess the patient’s current and achievable quality of life?	

Interview conclusion

All	· Is there anything we haven’t talked about yet, that you would like to say?	- Bring up unmentioned but interesting issues<br>- Lead participants out of the interview situation<br>- Thank you
